# Cogan’s Syndrome: Complex Diagnostics, Treatment, and Results of Hearing Rehabilitation in Long‐Term Follow‐Up—Case Series

**DOI:** 10.1155/crii/8559583

**Published:** 2025-12-22

**Authors:** Nóra Kecskeméti, Beáta Bencsik, Ágnes Szirmai, László Tamás, Marianna Küstel, András Grimm, Zsuzsanna Géhl, Hunor Sükösd, Péter Magyar, Anita Gáborján

**Affiliations:** ^1^ Department of Otorhinolaryngology, Head and Neck Surgery, Semmelweis University, Budapest, Hungary, petho.hu; ^2^ Department of Voice, Speech and Swallowing Therapy, Faculty of Health Sciences, Semmelweis University, Budapest, Hungary, petho.hu; ^3^ Department of Ophthalmology, Semmelweis University, Budapest, Hungary, petho.hu; ^4^ Medical Imaging Centre, Semmelweis University, Budapest, Hungary, petho.hu

**Keywords:** case series, cochlear implantation, Cogan’s syndrome, hearing loss

## Abstract

**Purpose:**

This study aims to present the diagnostic and therapeutic challenges of Cogan’s syndrome (CS) and the outcomes of hearing rehabilitation in long‐term follow‐up.

**Methods:**

Retrospective data analyses of patients with CS treated at Semmelweis University were performed. Comprehensive evaluations, including medical assessments, audiological measurements, otoneurological investigations, imaging, and ophthalmological examinations, were conducted on all patients.

**Results:**

Between 1995 and 2022, five patients with CS were followed. The severity and timing of ear and ocular symptoms varied. Bilateral, asymmetric hearing impairment manifested as sudden sensorineural hearing loss, and all patients experienced loss of bilateral vestibular function. Various ophthalmological manifestations showed instability over time. Systemic corticosteroids were the first‐line treatment, immunosuppressive therapy (methotrexate, cyclophosphamide, cyclosporin A), and biological treatment (infliximab, adalimumab) used as second‐ and third‐line therapies. Eye symptoms of all five patients were controlled by medications. For hearing impairment, four patients were treated with cochlear implantation and achieved long‐term stable speech perception. Hearing improvement was found in one patient by conservative therapy. One patient required reimplantation due to device failure, which was performed without complications.

**Conclusion:**

Sudden hearing loss and vestibular attacks in young patients require thorough investigation and close follow‐up. Early corticosteroid therapy or immunosuppressive and biological treatment can stabilize symptoms, including hearing levels. Early hearing rehabilitation with cochlear implants is crucial. Long‐term follow‐up indicates stable hearing levels and speech perception.

## 1. Introduction

In 1945, Cogan [1] described a syndrome characterized by nonsyphilitic interstitial keratitis (IK) and audiovestibular symptoms. Later, Cody and Williams [2] emphasized the systemic manifestations of this syndrome, now known as Cogan’s syndrome (CS).

CS is a rare chronic inflammatory disease that typically affects young adults with a mean age of 29 years and shows no sex preference [3, 4]. The typical form involves (i) ocular symptoms such as IK with or without conjunctivitis or iritis, (ii) progressive sensorineural hearing loss progressing to deafness within one to 2 months, and Meniére‐like vertigo attacks, and (iii) a less than 2‐year interval between hearing loss and ocular manifestations [3]. Atypical CS is characterized by audiovestibular symptoms with ocular manifestations other than IK, often associated with systemic vasculitis and a longer interval between symptom appearances, resulting in a worse prognosis [5]. Patients may also present with other organ symptoms such as neurological, gastrointestinal, or musculoskeletal issues [3, 6]. The combination of severe hearing loss and visual impairment significantly reduces the quality of life and leads to social isolation [7].

CS is considered an autoimmune disorder, with several studies supporting its autoimmune origin. Antibodies against the inner ear or cornea have been detected [8–11]. Some researchers suggest a correlation with systemic vasculitis based on clinical manifestations and ANCA‐positive cases [12–14]. Despite numerous studies, the etiology of CS remains unclear, making diagnosis challenging.

Corticosteroids, both topical and systemic, have been effective in treating IK and sudden hearing loss. However, corticosteroid‐resistant cases have been reported [15, 16]. For corticosteroid‐resistant patients, immunosuppressive treatments such as cyclosporin A, methotrexate, or azathioprine can be used [15, 17, 18]. Recent therapy options include TNF‐alpha blockers like infliximab and rituximab to control systemic effects and hearing loss [19, 20].

Prognosis and outcomes in patients with CS are unpredictable. Patients with severe hearing loss are at high risk of irreversible impairment; however, ocular manifestations generally have a good response rate to therapy [15]. Cochlear fibrosis and ossification can occur as early as 8 weeks after the initial symptoms due to inflammation in the inner ear [21]. Cochlear implantation is the only option for hearing rehabilitation in patients with progressive profound hearing loss [21–23].

The aim of this study is to present the diagnostic and therapeutic challenges of CS and the outcomes of hearing rehabilitation with cochlear implants and conventional hearing aids in long‐term follow‐up.

## 2. Materials and Methods

Data on patients with CS were collected at our clinic and collaborating clinics of Semmelweis University, where they were examined and treated. The retrospective study was approved by the Regional and Institutional Committee of Science and Research Ethics of Semmelweis University (Number: SE RKEB 124/2022). All patients underwent detailed examinations, including medical evaluation, subjective and objective audiological measurements, otoneurological investigations, temporal bone HRCT and MRI, ophthalmological investigations, and immunological, serological, and genetic blood tests.

### 2.1. Patients

Five patients (three females and two males) with hearing loss and dizziness diagnosed as CS between 1995 and 2022 were included. The diagnosis was always made by an immunologist. Their ages at the onset of cochleovestibular symptoms ranged between 20 and 31 years (average 26.8 years). Follow‐up time ranged from 6 months to 26 years. Patients were numbered (P1–P5) in order of the length of follow‐up time.

### 2.2. Audiological Investigations

The status of the outer and middle ear was checked by otomicroscopy and impedance measurement. Tympanometry was performed with a 226 Hz probe tone, and the stapedius reflex was checked using GSI Tympstar and Tympstar Pro equipment. Pure‐tone audiometric (PTA) evaluation was performed multiple times on all patients. Air conduction (AC) hearing threshold levels were measured at 125, 500, 1000, 2000, 4000, and 8000 Hz, and bone conduction (BC) threshold levels were recorded at 500, 1000, 2000, and 4000 Hz using a GSI 61 audiometer. Audiograms were compared over time. Distortion‐product otoacoustic emissions (DPOAE) were measured at 12 frequencies between 500 and 10,000 Hz using Interacoustics Titan Equipment. Auditory brainstem response (ABR) was measured using a click stimulus, and auditory steady‐state response (ASSR) was measured using GSI Audera and Interacoustics Eclipse. Speech audiometry was performed with Hungarian monosyllabic words when evaluable, and the efficiency of rehabilitation was measured in a free field with pure tones and speech tests.

### 2.3. Otoneurological Investigations

In cases of acute equilibrium complaints during the first otoneurological examination, usually conducted in the emergency department, the HINTS + examination was performed to differentiate between peripheral and central vestibular involvement in harmonic vestibular syndrome. The HINTS + examination includes the head–impulse test, spontaneous nystagmus investigation, eye skew deviation test, and tuning fork test [24]. This procedure has been used in the differential diagnosis of acute vestibular syndrome since 2009 but was not performed in all cases.

Later, as part of a detailed otoneurological examination, spontaneous and positional nystagmus and statokinetic tests such as Romberg and Unterberger–Fukuda were performed. The aim of our study was to examine the function of the vestibular end organ in CS patients. Electronystagmography (ENG) and Video‐Head–Impulse Test (VHIT) were also performed as part of the instrumental otoneurological investigations.

### 2.4. Ophthalmology

Slit‐lamp examinations were performed with a TOPCON ATE 600 slit lamp, and a VOLK superfield lens was used for fundus examination. Intraocular pressure was measured with Goldmann applanation tonometry.

### 2.5. Other Tests

Blood tests, immunological panels (ANA, ENA, ANCA), serological tests (*Treponema pallidum*, *Borrelia burgdorferi*, *Cytomegalovirus*, *Herpes simplex virus*, *Toxoplasma gondii*, etc.), cerebrospinal fluid examinations by lumbar puncture, and genetic tests (*GJB2* sequence analyses) were performed. Temporal bone HRCT and MRI scans were applied to exclude cochlear stenosis or fibrosis, central nervous system alterations and to plan cochlear implantation.

## 3. Results

Between 1995 and 2022, five patients with CS were diagnosed and treated in the Department of Otorhinolaryngology, Head and Neck Surgery at Semmelweis University. In all cases, the diagnosis of CS was made by immunologists after the onset of cochleovestibular symptoms. The accompanying inflammation of the eyes confirmed the diagnosis. The severity and timing of symptoms varied among the patients (Table 1).

**Table 1:** Types and severity of symptoms, the applied treatment and hearing rehabilitation in our patients.

Patient	Age at diagnosis (year)	Symptoms				Eye‐ear symptom‐interval	Progression of hearing loss	Conservative treatment		Hearing rehabilitation	
		Audiovestibular	Ocular	Immunoserological tests	Other			Local	Systemic	Type	Time to surgery
P1	26	Bilateral acute profound HL	Keratouveitis	ANA − ENA − ANCA −	–	3 months	no	—	Corticosteroid	CI l.d.	6 months
P2	31	Bilateral acute mild‐to‐moderate HL, tinnitus, vertigo	Relaptiform keratitis, uveitis, scleritis bilat.	ANA + ENA − ANCA −	–	1 year	To profound HL in a few weeks	IT dexamethasone, steroid eyedrops	Corticosteroid, methotrexate, adalimumab	CI l.d.	2 years
P3	29	Unilateral mild HL, tinnitus, vertigo	Interstitial keratitis, keratoconjunctivitis	ANA + ENA − ANCA −	Abdominal pain	3 days	To profound HL in weeks on left side, moderate HL in 6 months on right side	IT dexamethasone, steroid eyedrops	Corticosteroid (1,2 year long), Cyclophosphamide, infliximab	CI l.s.	10 months
										HA l.d.	—
P4	20	Deafness l.d, moderate‐severe HL l.s., vertigo	Granulomatosus anterior uveitis	ANA + ENA − ANCA −	Weight loss	Same time	No	Steroid eyedrops	Corticosteroid	CI l.d.	6 weeks
										HA l.s.	—
P5	28	Bilateral moderate HL	Interstitial keratitis, panuveitis, frosted branch angiitis	ANA + ENA − ANCA −	Fever, diarrhea, erythema, arthralgia, myalgia	few days	To moderate‐profound HL l.s in a few weeks.	IT Dexamethasone, steroid eyedrops	Corticosteroid, methotrexat	HA l.u.	—

*Note:* l.d. = right side, l.s. = left side, l.u. = bilateral.

Abbreviations: CI, cochlear implantation; HA, hearing aid; HL, hearing loss; IT, intratympanic.

Initial symptoms were highly diverse. P1 presented with sudden bilateral hearing loss, with eye symptoms appearing 3 months later. P2 had progressive bilateral hearing loss with vertigo and tinnitus, with eye symptoms developing within 1 year. P3 initially had eye symptoms, followed by sudden sensorineural hearing loss with tinnitus and vertigo 3 days later. P4 had eye symptoms and sudden hearing loss simultaneously. P5 experienced diarrhea, fever, articular pain, and eyelid edema, followed by progressive sensorineural hearing loss and severe vision complaints within a few weeks. Symptoms started 3 days after an mRNA‐based SARS‐CoV‐2 vaccination. Immunoserological tests showed ANA positivity in three patients (P2, P3, and P4); ENA and ANCA were negative in all patients. CRP was elevated in one patient only (P5); she had severe systemic inflammation at the initial part of the disease.

**Figure 1:**
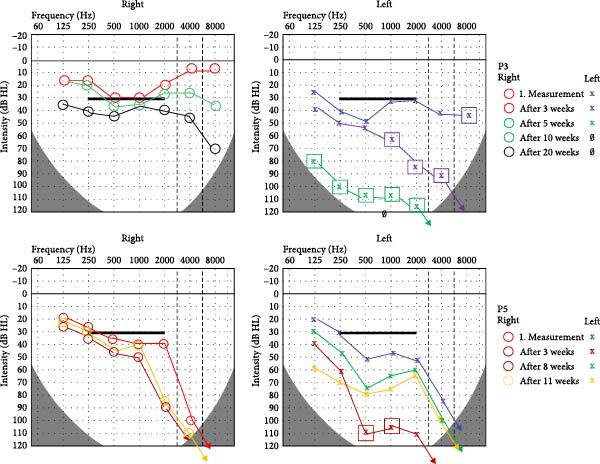
Pure tone audiograms for patients with progressive (P3) and fluctuating (P5) sensorineural hearing loss (HL). In P3, sudden hearing loss progressed over 10 weeks and showed stability despite therapy after 20 weeks on left side. P5 had fluctuating sensorineural hearing loss on the left side, with profound HL at Week 8, showing significant improvement after methotrexate and intratympanic dexamethasone treatment (yellow line, the color of the hearing test curves was not changed if the measurement results taken at different times were the same).

Hearing impairment was bilateral in all patients at some point during follow‐up, presenting as sudden sensorineural hearing loss. The lack of otoacoustic emission, Metz recruitment, narrowing of the dynamic range, and roll‐over in speech understanding tests confirmed the inner ear origin. Hearing loss was asymmetric in four patients. The loss of function was stable and irreversible in two patients (P1 and P2). P2 experienced sudden profound hearing loss on the right side first, followed by the left side 2 months later. Unilateral sudden hearing loss with rapid progression to deafness and stable mild to moderate HL on the other side was observed in two patients (P3 and P4, Figure 1). The hearing impairment of P5 fluctuated depending on the therapeutic regimen. Profound sensorineural HL was measured after 8 weeks, with significant improvement after 2 weeks of intratympanic corticosteroid and combined methotrexate therapy (Figure 1).

**Figure 2:**
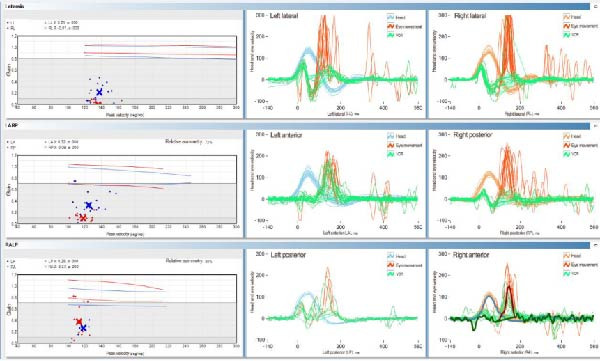
Results of VHIT in P5 showing loss of vestibular function, decreased gain, and refixation saccades in all semicircular canals. Normal gain values are higher than 0.8 in horizontal canals and higher than 0.7 in vertical canals. Patient gain values: right lateral: 0.01, left lateral: 0.21, left anterior: 0.32, right posterior: 0.09, right anterior: 0.37, and left posterior: 0.26. The red spikes on the graphics show the refixation of the eyeballs, due to the corrective function of the brainstem. The VHIT (video head impulse test) measures the function of the three semicircular canals of the inner ear’s balance organ (vestibular system). This test is based on the vestibulo–ocular reflex (VOR), which is a reflex that acts to stabilize gaze during head movement. If the VOR is not working properly, individuals may experience blurred or bouncing vision (oscillopsia), dizziness, and imbalance.

Vestibular functions were tested at scheduled appointments. Mild ataxia was found in four patients without spontaneous symptoms. The provocation tests (VHIT and caloric test) evaluated bilateral loss of function in all cases (Figure 2). P3 had unilateral loss of function at the first examination, with acute left caloric weakness. After slow central compensation, 6 months later, the patient again experienced vertigo complaints, and the right labyrinth loss of function was confirmed. Thus, bilateral loss of the vestibular end organ occurred at different times for P3, which is unusual.

Ophthalmological investigations showed different types of ocular manifestations. P1 had keratouveitis on the left side with normal vision, treated with topical corticosteroids. P2 suffered from bilateral recurrent keratitis, scleritis, and uveitis. Due to severe symptoms, methotrexate and systemic corticosteroid therapy were initiated. 6 years after the first symptoms, the patient developed secondary cataracts bilaterally and underwent phacoemulsification with artificial lens implantation. P3 had conjunctivitis with IK. P4 had granulomatous anterior uveitis on the left side, which became bilateral after a few months. Systemic and topical corticosteroid therapy was applied, with symptoms still fluctuating. P5 had bilateral IK, panuveitis, and frosted branch angiitis. Symptoms resolved after systemic immunosuppressive therapy.

In patients P2 and P3, negative results of sequence analysis of the *GJB2* gene ruled out the most frequent genetic origin. Serological tests were negative in all patients. Due to severe systemic symptoms, lumbar puncture was performed in patients P3 and P5, also with negative results. Colonoscopies were performed on three patients, and the histology ruled out Chron’s disease in every case.

CT scans of the temporal bone were performed in all patients, and MRI scans of the inner ear in four patients. P1 was implanted in 1998 when MRI was not yet routinely used in preoperative imaging protocols. No obliteration or fibrosis was found on the scans.

The initial treatment regimens were the same for all patients. Initially, systemic corticosteroids were administered intravenously in high doses (methylprednisolone 250 mg/day on days 1–3, 125 mg/day on Days 4–6), followed by oral administration for 15 days. In some patients, corticosteroids were administered orally at doses of 0.5–1 mg/kg body weight daily for longer periods, even up to years (P3), to control vertigo attacks and eye symptoms. Local corticosteroid treatment was also administered in four patients. P2, P4, and P5 received intratympanic corticosteroid treatment (dexamethasone 4 mg/day for 5 days) and corticosteroid eye drops (dexamethasone or prednisolone). P3 received topical corticosteroid for eye symptoms only. Despite corticosteroid treatment, P2, P3, and P5 had uncontrolled disease and were treated with methotrexate or cyclophosphamide. In two cases, biological treatment was required, and the TNF‐alpha inhibitors adalimumab (P2) and infliximab (P3) were started to control severe symptoms with good efficacy (Table 1).

Hearing rehabilitation was crucial after the initial period of hearing loss. Cochlear implantation was applied in four patients, all unilaterally implanted. Three patients received conventional hearing aids to improve residual hearing (Table 1). The time between onset and implantation varied from 6 weeks to 2 years. In one case, implantation was performed 2 years after sudden hearing loss because the patient was hesitant to undergo surgery. Neither obliteration nor stenosis of the cochlea was found during surgery, and the implantations were uneventful. The CIs were one MedEl, two Nucleus, and one Advanced Bionics. Postoperative electrode extrusion was observed in one patient (P4), necessitating reoperation and electrode repositioning in the early postoperative period immediately after implantation. Extrusion was confirmed by postoperative radiography, routinely performed on the first postoperative day. No complications were observed after the second insertion, and radiography revealed that the electrode was in the correct position. In P1, the electrode was implanted through the posterior wall of the external ear canal under the eardrum (the surgical technique at the time). In this patient, the electrode was pulled out unexpectedly during routine ear cleaning; therefore, explantation and reimplantation were performed 5 years after the first surgery. Postoperative fittings were uneventful, and all patients had good speech perception after the first fitting. In three patients, a conventional hearing aid was added to the nonimplanted side, improving binaural hearing. In P5, moderate to severe fluctuating hearing loss was observed, and the patient does not currently require surgical hearing rehabilitation.

Follow‐up varied from 6 months to 25 years. Long‐term follow‐up after implantation ranged from 1.5 to 24 years. Speech processors are regularly upgraded every 6 years. Speech perception remains stable in three patients who have used the processor for more than 2 years. No fluctuations in the impedances of the electrodes were measured. P4 has problems with speech perception and lower compliance, missing regular training sessions.

## 4. Discussion

In our case series, the sex ratio, age of onset, and audiovestibular and ocular symptoms were similar to other CS studies [3–6]. The diagnostic criterion for the typical disease was nonsyphilitic IK with audiovestibular symptoms within 2 years, observed in four patients. One case was considered atypical due to eye symptoms other than IK; however, the distinction between typical and atypical forms is not always clear.

Hearing impairment typically presents as sudden‐onset bilateral sensorineural hearing loss, often fluctuating, with 60% of cases progressing to profound hearing loss without appropriate treatment [25]. The audiological characteristics often mimic other sudden hearing losses, potentially delaying correct diagnosis and early treatment. In our cases, asymmetric hearing loss was observed in four patients. There was one case of bilateral sudden profound hearing impairment, with bilateral deafness observed in another case 2 months apart. Unilateral acute hearing loss with unilateral acute vestibular dysfunction was primarily observed in one patient, with bilateral vestibular loss of function developing over a few months. In one case, progression to deafness occurred despite corticosteroid therapy, while fluctuation was detected in another case depending on the treatment type. Overall, 80% of patients had deafness on at least one side, and 40% had bilateral deafness, a high ratio demanding attention in young patients. Differential diagnosis from other cochleovestibular diseases is often not easy. The characteristic of the vestibular loss of function and hearing loss can help. Warning signs for diagnosis of CS could be the sudden, progressive hearing loss in young patients (under 30 years of age), the vertigo attacks lasting for days or even weeks (in *M. Meniére* the attacks typically last from 20 min to 4 h with tinnitus and hearing loss, in neuritis the vertigo usually lasts for days without hearing loss), and the eye symptoms.

Treatment varies among patients. Ocular symptoms responded well to corticosteroids alone, disease‐modifying antirheumatic drugs and biological treatments [15]. A French nationwide study indicated infliximab alone had the best impact on audiovestibular symptoms [19]. In our cases, most ocular symptoms were uncontrolled with topical corticosteroid treatment, leading to the introduction of immunosuppressive therapy with methotrexate or cyclophosphamide. One patient developed a cataract due to long‐term corticosteroid use. Biological treatment was required in two nonresponding cases, where adalimumab and infliximab effectively controlled ocular and vestibular manifestations. Hearing improvement was observed in one case, with methotrexate and intratympanic steroid usage achieving significant improvement. Intratympanic steroid is often used in sudden sensorineural hearing loss as an alternative option for systemic corticosteroid treatments [26]. Dexamethasone is administered through the tympanic membrane, directly into the middle ear. The advantage of intratympanic steroid treatment is that it lasts a shorter time, and there are no systemic corticosteroid side effects. It can be effective in autoimmune inner ear disease as well [16]. Four other cases with profound hearing loss did not respond to any treatment, resulting in cochlear implantation. In one case with asymmetric hearing loss, infliximab controlled the better hearing ear. Infliximab was introduced 3 months after the hearing loss appeared in the better hearing ear (the other ear was deaf and implanted previously), suggesting infliximab as an early therapy option.

Hearing rehabilitation is crucial for ensuring good speech perception and communication in daily life. Conventional hearing aids and/or cochlear implants can be applied depending on the severity of hearing loss. Rehabilitation with conventional hearing aids is a good alternative for mild to severe hearing loss; however, close collaboration with an audiologist is required due to fluctuating hearing levels. Early cochlear stenosis and fibrosis can develop in CS, appearing as early as 8 weeks after the onset of profound hearing loss, indicating irreversibility [21]. Fibrosis can be excluded by MRI and ossification by HRCT of the temporal bone. Stenosis can complicate electrode insertion into the cochlea, so early imaging and implantation are crucial. Cochlear implantation has no effect on cochlear ossification and disease progress, but in opposite, the ossification may make subsequent implantation impossible because the electrode cannot be inserted. There are various middle ear diseases, such as cholesteatoma, where it is routinely necessary to perform control MRI examinations after surgery to control the recurrence of the disease [27]. There is no such recommendation for inner ear diseases, although repeated MRI examinations may be recommended in CS in cases of progressive hearing loss as well. In this study, neither stenosis nor obliteration was observed. One patient received a cochlear implant 2 years after sudden hearing loss without intraoperative complications, and another patient requiring reimplantation also had no difficulties. Cochlear implant users show good speech perception results in CS. Long‐term follow‐up showed no fluctuations in impedances or hearing levels, unlike post‐meningitis patients who often experience cochlear fibrosis and ossification [28].

In one case, vaccination may have triggered CS. There are some publications already, which suggests that COVID‐19 vaccination can provoke autoimmune diseases in susceptible individuals [29]. One article suggest mRNA vaccination as a trigger factor for autoimmune vestibulopathy [30]. However, this claim requires further research to establish any correlation.

Sudden hearing loss and vestibular attacks in young patients, even if unilateral, require thorough investigation and close follow‐up. In cases of eye symptoms, redness, fluctuating and progressive hearing loss, or vertigo attacks, a possible autoimmune origin should be considered. Early imaging can assist in both diagnosis and hearing rehabilitation. Early therapy with corticosteroids or immunosuppressive and biological treatments can stabilize symptoms, including hearing levels. For deafened patients, early hearing rehabilitation with cochlear implants is crucial due to potential cochlear ossification. These patients should be managed in centers with close teamwork between immunologists, otorhinolaryngologists, ophthalmologists, and radiologists.

## Conflicts of Interest

The authors declare no conflicts of interest.

## Funding

No funding was received for this manuscript.

## Data Availability

The research data are not shared.
